# The Rab3 family proteins in age-related neurodegeneration: unraveling molecular pathways and potential therapeutic targets

**DOI:** 10.1038/s41514-025-00257-6

**Published:** 2025-07-14

**Authors:** Haijun He, Ruixue Ai, Evandro Fei Fang, Konstantinos Palikaras

**Affiliations:** 1 Department of Physiology, Medical School, National and Kapodistrian University of Athens, 157 27, Athens, Greece; 2 Department of Clinical Molecular Biology, University of Oslo and Akershus University Hospital, 1474 Lørenskog, Norway; 3 The Norwegian Centre on Healthy Ageing (NO-Age) and the Norwegian National Anti-Alzheimer’s Disease (NO-AD) Networks, 0372 Oslo, Norway

**Keywords:** Autophagy, Neural ageing, Neurodegeneration

## Abstract

The Rab3 protein family is composed of a series of small GTP-binding proteins, including Rab3a, Rab3b, Rab3c, and Rab3d, termed Rab3s. They play crucial roles in health, including in brain function, such as through the regulation of synaptic transmission and neuronal activities. In the high-energy-demanding and high-traffic neurons, the Rab3s regulate essential cellular processes, including trafficking of synaptic vesicles and lysosomal positioning, which are pivotal for the maintenance of synaptic integrity and neuronal physiology. Emerging findings suggest that alterations in Rab3s expression are associated with age-related neurodegenerative pathologies, including Alzheimer’s disease, Parkinson’s disease, and Huntington’s disease, among others. Here, we provide an overview of how Rab3s dysregulation disrupts neuronal homeostasis, contributing to impaired autophagy, synaptic dysfunction, and eventually leading to neuronal death. We highlight emerging questions on how Rab3s safeguards the brain and how their dysfunction contributes to the different neurodegenerative diseases. We propose fine-tuning the Rab3s signaling directly or indirectly, such as via targeting their upstream protein AMPK, holding therapeutic potential.

## Introduction

### Overview of age-related neurodegenerative diseases and their socio-economic impacts

Age-related neurodegenerative diseases are a group of disorders characterized by the progressive degeneration of the structure and function of the nervous system, which finally leads to brain dysfunction, especially memory loss. These conditions include Alzheimer’s disease (AD), Parkinson’s disease (PD), Huntington’s disease (HD), among others^1,2^. Patients experience cognitive and motor decline, leading to a gradual loss of independence and reduced quality of life, while families and caregivers endure significant emotional and physical stress. Thus, they impose a profound and multifaceted burden on individuals and society^3,4^.

The global aging population has driven to a significance rise in the prevalence of these diseases, transforming them into a major public health challenge^4–7^. The societal impacts extend beyond individual health, including considerable economic burdens, encompassing direct medical expenses, long-term care costs, and productivity loss. Simultaneously, family caregivers face a combination of emotional stress and financial burdens, underscoring the urgent need for research into the underlying mechanisms of neurodegenerative diseases and the development of effective therapeutic strategies^4,5^.

### Importance of Rab3 proteins in neuronal function and synaptic transmission

**Fig. 1:**
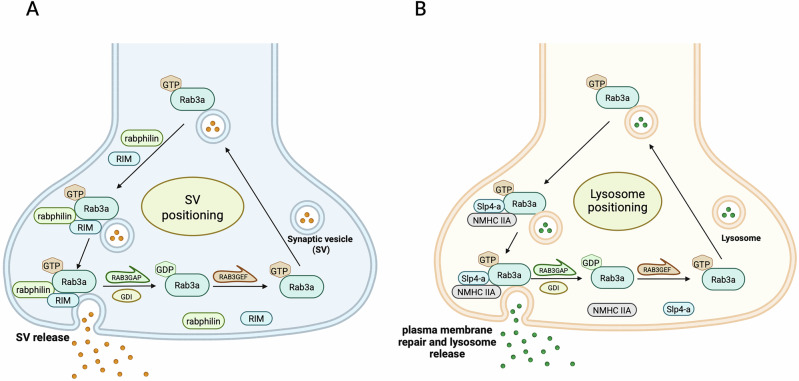
Rab3a cycle of synaptic vesicle and lysosome positioning. **A** GTP-bound Rab3 on the vesicle membrane interacts with effector proteins such as rabphilin and the Rab3-interacting molecule (RIM), facilitating the docking of synaptic vesicles and the subsequent release of neurotransmitters. Following exocytosis, GTP-Rab3 is hydrolyzed to its GDP-bound form. The GDP dissociation inhibitor (GDI) subsequently binds to GDP-Rab3, thereby dissociating it and its associated effectors from the vesicle membrane. The soluble GDP-Rab3 can then re-associate with the vesicle membrane upon the action of guanine nucleotide exchange factors (GEFs), which catalyze the conversion of GDP to GTP. **B** The mechanism of lysosome positioning facilitated by Rab3a parallels that of synaptic vesicles. Upon recruitment by Rab3a, the lysosome forms a complex with Rab3a, Slp4a, and NMHC IIA. This complex is essential for positioning lysosomes near the cell membrane, thereby promoting their fusion with the compromised membrane and playing a vital role in the membrane repair process. Created in BioRender. Palikaras, K. (2025) https://BioRender.com/t13j594.

RAB (Ras-associated binding) are small molecular switches of about 200–250 amino acids that are activated by guanine nucleotide exchange factors (GEFs) and deactivated by GTP hydrolysis activator proteins (GAP)^8–11^. Rab3 proteins, including four isoforms in mammals called Rab3a, Rab3b, Rab3c, and Rab3d, are small GTP-binding proteins that play a crucial role in the regulation of synaptic vesicle trafficking and neurotransmitter release^12^. These proteins share 77–84% amino acid sequence identity, with the greatest divergence in their N-terminal and C-terminal regions^13,14^. They are predominantly associated with synaptic vesicles in neurons and are essential for maintaining efficient synaptic transmission. Rab3 proteins are involved in the final stages of synaptic vesicle exocytosis, a process critical for neurotransmitter release. They help in the docking and priming of synaptic vesicles at the presynaptic membrane, ensuring that neurotransmitters are released in response to calcium influx. GTP-Rab3 on the vesicle membrane can bind to effector proteins, such as rabphilin and Rab3 interacting molecule (RIM)^15^, aiding in the docking of synaptic vesicles^16–18^ and release of neuropeptide and neurotrophin^19^. After exocytosis, GTP-Rab3 converts to its GDP-bound form and GDP dissociation inhibitor (GDI) then binds to GDP-Rab3, detaching it and its effectors from the vesicle membrane^20,21^. Subsequently, the soluble GDP-Rab3 can reattach to the vesicle membrane when GEF converts GDP to GTP^22,23^ (Fig. 1A).

Among the four isoforms of Rab3 protein family, Rab3a, Rab3b, and Rab3c are mainly found in neurons^24–26^, whereas Rab3d is mostly present in non-neuronal cells like the parotid gland, pancreas, and mast cells, and is involved in the maturation and secretion of secretory granules^27–29^. Rab3a, the most abundant and well-studied isoform in synaptic vesicles, regulates vesicle transport, docking, fusion, and Ca^2+^-dependent neurotransmitter release by transitioning between GDP-bound and GTP-bound states and interacting with other synaptic proteins^8,30,31^. Deleting all Rab3 genes in mammalian neurons significantly hindered vesicle fusion, but reintroducing Rab3a restored this function, highlighting Rab3a’s crucial role in vesicle fusion^19^. These proteins are also involved in maintaining the pool of synaptic vesicles available for release. Mutations or deficiencies in Rab3 proteins can lead to a reduction in the number of synaptic vesicles at active zones, impairing synaptic transmission. Knocking down Rab3a or injecting antisense oligonucleotides into PC12 cells significantly boosts Ca^2+^-triggered dopamine release and increases secretory activity in bovine adrenal chromaffin cells^32–36^. Conversely, elevating intracellular Rab3a, either by overexpression or adding Rab3a protein to permeabilized PC12 cells, inhibits Ca^2+^-dependent neurotransmitter release^35–37^. Taken together, these findings show that overexpressing Rab3a in its GTP-bound state, but not in its GDP-bound state, leads to the loss of secretory vesicles and inhibits exocytosis^32,35^. This phenomenon suggests that Rab3a does not inhibit secretion directly but rather depletes dense core vesicle content by driving continuous exocytosis. This notion is further supported by evidence indicating that Rab3a, when permanently bound to GTP, activates the continuous secretion of co-transfected human growth hormone (hGH), thereby reducing its intracellular levels^35^. Additionally, neurotransmitter release occurs through both Ca²⁺-independent and Ca²⁺-dependent pathways, with Rab3a playing a role in regulating both via distinct mechanisms^36^.

Emerging findings suggest that Rab3a may modulate neurotransmitter release by interacting with synaptotagmin proteins^38,39^, which are pivotal regulators of Ca^2+^-dependent neurotransmitter release^31^. Synaptotagmin proteins play a crucial role in regulating various biological processes, particularly in mediating synaptic vesicle fusion with the target plasma membrane, as well as in facilitating both exocytosis and endocytosis^40–44^. The knockdown of synaptotagmin and the microinjection of anti-synaptotagmin antibodies or synaptotagmin fragments into living cells lead to a reduction or complete ablation of neurotransmitter release^36,45,46^.

Synaptotagmin 1 (Syt1), characterized by its two principal functional domains, C2A and C2B, is the predominant isoform and the most extensively studied among the 17 known synaptotagmin isoforms^47^. Synaptotagmin, a Ca^2+^-binding vesicular membrane protein, is essential for vesicle fusion and Ca2+-triggered neurotransmitter release, whereas Rab3 limits the number of vesicles fusing with the plasma membrane during Ca^2+^ influx, resembling the yin and yang balance in ancient Chinese philosophy^31,38^. Recent studies indicate that Syt1 maintains Rab3 in its GTP-bound state by binding to and inhibiting the GAP catalytic subunit RBG-1 on the vesicle membrane, a process dependent on Ca^2+^ influx^48^. Without Ca^2+^, Syt1 binds RBG-1, preventing GTP hydrolysis of Rab3 and keeping it membrane-bound. When Ca^2+^ levels rise due to an action potential, Syt1 binding to RBG-1 is disrupted, allowing Rab3 to hydrolyze GTP to GDP, leading to the release of Rab3 from the membrane and its effectors^48^.

This suggests that Syt1 promotes the recycling of Rab3a from synaptic vesicles following exocytosis, thereby supporting efficient vesicle turnover and sustained neurotransmission. However, Rab3a can also act through a distinct, inhibitory mechanism to modulate synaptic release.

Rab3a exists in two states: a soluble, GDP-bound form and a membrane-associated, GTP-bound form. In vitro studies have shown that the soluble (GDP-bound) form of Rab3a can interfere with Syt1 function by competitively inhibiting its interactions with key synaptic components. Specifically, Rab3a disrupts the binding of the C2B domain of Syt1 to syntaxin, and the binding of the C2A domain to phospholipids^39,49^. Since Syt1 serves as a primary Ca²⁺ sensor during synaptic vesicle fusion—mediating Ca²⁺-dependent binding to phospholipids and SNARE proteins such as synaptobrevin/VAMP, syntaxin, and SNAP-25^50^. This interference by Rab3a suggests a regulatory feedback mechanism.

By modulating Syt1’s interactions with membrane lipids and SNARE proteins, Rab3a may act to fine-tune neurotransmitter release, potentially preventing excessive or premature exocytosis^38^. Therefore, the Rab3–Syt1 interaction represents a key regulatory axis that not only coordinates synaptic vesicle cycling but also ensures precise control of Ca²⁺-dependent synaptic transmission.

Rab3 activity is tightly regulated by a balance between guanine nucleotide exchange factors (GEFs), which promote the active GTP-bound state, and GTPase-activating proteins (GAPs), which facilitate GTP hydrolysis to return Rab3 to its inactive GDP-bound state^8–11^. Two well-characterized Rab3 GEFs are MADD (also known as Rab3GEP or DENN) and GRAB (also referred to as Rab3IL1), with differing substrate specificities, while Rab3-GAP is composed of Rab3GAP, TBC1D10B, and USP6NL^51–55^.

MADD functions as a GEF for all four Rab3 isoforms (Rab3a-d) in neuronal, neuroendocrine, and endothelial cells, as well as for Rab27A in melanocytes, parotid acinar cells, and endothelial cells^53,56–61^. In contrast, GRAB exhibits more selective activity and has been shown to specifically activate Rab3a^55^, suggesting the existence of isoform-preferential regulation.

On the inactivation side, the Rab3GAP complex, which consists of the catalytic subunit Rab3GAP1 and the non-catalytic subunit Rab3GAP2, acts as a GAP for all Rab3 isoforms^54,62^ and as a GEF for Rab18^63^, indicating functional versatility. Genetic mutations in *Rab3GAP1* cause Warburg Micro syndrome, a rare autosomal recessive disorder characterized by severe neurodevelopmental defects, ocular anomalies and hypogonadism^64^. Mutations in *Rab3GAP2* lead to Martsolf syndrome, a phenotypically milder condition that shares overlapping features with Warburg Micro syndrome^65^.

Beyond the Rab3GAP complex, several TBC-domain-containing proteins (TBC/RABGAPs) have been identified with broader Rab specificity^66^. TBC1D10B (also known as FLJ13130) has GAP activity toward Rab3a, Rab22A, Rab27A, Rab31, and Rab35^6^, while USP6NL (also known as RN-TRE) acts on Rab3a as well as Rab1, Rab2, Rab5a, Rab28, Rab41, and Rab43^67,68^. These GAPs are not Rab3-specific but are capable of modulating Rab3a among other Rab GTPases.

Taken together, current evidence indicates that while some regulators such as Rab3GEP and Rab3GAP act broadly across Rab3 isoforms, others such as GRAB and certain TBC-domain GAPs show isoform-selectivity or broader substrate promiscuity. This suggests a layered regulatory mechanism where pan-Rab3 and isoform-specific control may coexist, potentially allowing cell-type or context-dependent fine-tuning of Rab3 signaling.

### Rab3 associates with lysosomes and autophagy

The lysosome, first discovered by Nobel laureate Christian de Duve in the 1950s, is the central digestive organelle in almost all eukaryotic cells. As the final component of the endocytic pathway, it plays a vital role in numerous biological processes, including endocytosis, exocytosis, macropinocytosis, plasma membrane repair, pathogen defense, cell death, signal transduction, and autophagy^69–72^. Emerging evidence suggests that different isoforms of Rab3 proteins are actively involved in the regulation of lysosomal function and autophagy, underlining their significance in cellular homeostasis.

Several studies have shed light on the role of Rab3a in lysosomal exocytosis and plasma membrane repair, revealing that silencing Rab3a leads to the clustering of near the cell nucleus and a corresponding inhibition of plasma membrane repair^73–75^. These findings suggest that beyond its established role in synaptic vesicle dynamics, Rab3a also facilitates lysosome recruitment and positioning. Following recruitment, Rab3a interacts with lysosomes to form a protein complex involving Slp4a and NMHC IIA. This complex is crucial for positioning lysosomes in close proximity to the cell membrane, facilitating their fusion with the damaged membrane and contributing to efficient membrane repair^73^ (Fig. 1B). In addition to Rab3a, other Rab3 isoforms are emerging as key regulators in autophagy and cellular homeostasis. Notably, an integrated analysis of extrachromosomal circular DNA (eccDNA) sequencing and transcriptome data implicated Rab3b in autophagy regulation. These studies suggest that Rab3b might be transcribed from eccDNA and could play a significant role in conferring cisplatin resistance in cancer cells by inducing autophagy^76^. These findings underscore the potential of Rab3b as a therapeutic target in overcoming drug resistance. Moreover, the deficiency of Rab3d, coupled with conformational alterations in GOLGA4, has been found to significantly influence MYH9-dependent ethanol-induced Golgiphagy^77^. Golgiphagy is a selective type of autophagy targeting the Golgi apparatus for degradation, playing a critical role in cellular adaptation to stress^77^. The contrasting effects of Rab3b and Rab3d on autophagy suggest that different isoforms of Rab3 proteins may have opposing roles in this process, necessitating further investigation to elucidate the underlying mechanisms.

In addition to studies conducted on mammals, research involving *Drosophila melanogaster* has demonstrated that the inactivation of the small GTPase Rab3 replicates the phenotypic presynaptic Arl8 accumulation observed in *LRRK2* mutations^78^. Arl8, a regulator of lysosomal fusion and motility, functions as a cellular marker for lysosomes within the cell body and as an indicator for presynaptic lysosome-related vesicles (PLVs) at presynaptic sites^79^. In mammals, Rab3 proteins, as well as other Rab proteins like Rab1a/b, Rab5a/b/c, Rab8a/b, Rab10, Rab12, Rab29, Rab35, and Rab43 are reported to be specifically phosphorylated by *LRRK2*^80,81^. Among these, Rab3, Rab5, Rab8, Rab10, Rab29, and Rab35 have been documented to exhibit correlations with lysosomal functions and autophagic processes^73,76,77,82–89^. In *D. melanogaster*, Arl8-positive structures exhibit significant co-localization with Rab3, while demonstrating minimal co-localization with Rab10 and Rab35^78^. Additionally, there is an absence of Rab8 and Rab32 (the human Rab29 ortholog) signals within the Arl8-positive structures^78^. This suggests that Arl8 exhibits a higher specificity towards Rab3 proteins in flies, and it is plausible that the function of Rab3 in lysosomal activity is significantly influenced by its interaction with Arl8. Meanwhile, both lysosomes and PLVs share the common feature of being acidic organelles, and Arl8 is believed to be involved in their transport^78^. The present study does not elucidate whether the presynaptic accumulation of Arl8 is linked to alterations in the autophagy-lysosome pathway. However, considering that Arl8 accumulation partially co-localizes with Endophilin A (EndoA), it is plausible that this accumulation may interfere with EndoA function, thereby further impairing EndoA-mediated autophagy at the presynaptic sites in flies with *Lrrk2* mutations^78,90^.

Beyond Rab3 proteins, many members of the Rab protein family are involved in regulating lysosomal function and associated cellular processes. RAB2 and RAB14 play a regulatory role in the formation of autophagosomes and autolysosomes^91–93^. RAB5 has been identified as a regulator of autophagy in a growing number of cellular processes, acting upstream of LC3 conjugation, a mechanism referred to as “RAB5-regulated autophagy”^82,87,94^. Rab7 is a key regulator of lysosome biogenesis, late endosome-lysosome fusion, and lysosomal transport, playing a central role in the maturation of endosomes and lysosomes^86,95,96^. Similarly, impairments in Rab8a or Rab10 function result in endolysosomal trafficking deficits that mirror those observed with the pathogenic G2019S mutation in *LRRK2*, a mutation linked to Parkinson’s disease, and are accompanied by a diminished Rab7 activity^83^. The Rab9-dependent autophagosome plays a crucial role in inducing alternative autophagy, which operates independently of Atg5 and Atg7^97–99^. Rab26 is known to promote the perinuclear aggregation of lysosomes, which in turn triggers the redistribution of mitochondria^100^, while the knockout or knockdown of Rab29 disrupts retrograde trafficking between the late endosome and Golgi complex, impairing lysosomal enzyme transport and lysosomal homeostasis^84,85,88^. Rab27b enhances lysosomal function and facilitates the clearance of α-syn in neuronal cells^101^. Rab32 associates with lysosomes and regulates cellular proliferation and metabolism by facilitating mTORC1 signaling^102^. Rab35, a regulator of endocytosis, has also been implicated in facilitating the targeting of NDP52 to damaged mitochondria during the process of mitophagy^89^.

In addition to the role in regulating cellular vesicle transport mechanisms, numerous RabGAP proteins containing TBC (TRE2-BUB2-CDC16) domains have recently been implicated in autophagic processes^103–106^. Genetic studies in *Caenorhabditis elegans* and human primary fibroblasts reveal that the Rab3GAP complex, consisting of Rab3GAP1 and Rab3GAP2, modulates autophagy and protein aggregation under both basal and rapamycin-induced conditions, even though these proteins lack a TBC domain^107^. While the role of Rab3 proteins in synaptic transmission has been extensively studied, their connection to lysosomal function warrants further investigation. Notably, Rab26 overexpression leads to lysosomal aggregation in the perinuclear region and subsequently to mitochondrial redistribution^100^, a phenomenon reminiscent of the effects observed when Rab3a is silenced. Silencing Rab3a prompts lysosomes to cluster near the cell nucleus, suggesting that Rab3a might influence lysosomal positioning and autophagy^73–75^. Taken together, these findings underline the diverse and isoform-specific functions of Rab3 proteins, potentially linking lysosomal function to organelle reorganization and cellular homeostasis.

### The phenotypes of Rab3 mutants across species

Given the essential role of Rab3 in synaptic vesicle function, various Rab3 mutant models have been developed in mice^12^, *C. elegans*^16^, and *D. melanogaster*^108^ to investigate its function in vivo. Mammals possess four distinct Rab3 isoforms, Rab3a, Rab3b, Rab3c, and Rab3d, with distinct yet overlapping neuronal expression pattern^35^. Knockout (KO) mice lacking two or three Rab3 isoforms remain viable, whereas quadruple KO mice, deficient in all four, die shortly after birth due to respiratory failure^12,109^. Despite normal brain morphology and synaptic architecture, these mice show severely attenuated synaptic responses, underscoring the essential role of Rab3 in neurotransmission^12^. Among the four isoforms, Rab3a appears to be particularly critical. Triple KO mice lacking Rab3b, Rab3c and Rab3d (BCD −/− mice) remain viable, even with only one functional Rab3a allele, suggesting that Rab3a is the most indispensable for survival^12^. This suggests that Rab3a plays a more critical role in the survival of mice compared to the other three Rab3 genes. In Rab3a KO mice, long-term potentiation (LTP) at hippocampal mossy fiber synapses is eliminated, potentially leading to deficits in learning and memory^110^. These animals also show moderately impaired performance in spatial and episodic-like memory tasks in the water maze and mild deficits in spatial working memory in the radial maze^111^. Furthermore, synaptic transmission is markedly depressed following brief repetitive stimulation (15–30 stimuli at 14 Hz)^112^, although exocytosis per synapse is not impaired^33^. Interestingly, quantal release per synapse is selectively increased in the hippocampus, indicating a role for Rab3a in fine-tuning synaptic output. Rab3b KO mice show disruption of the endocannabinoid-dependent long-term depression but maintain normal NMDA receptor-dependent LTP^113^. While overall learning and memory appear unaffected, these mice exhibit improved reversal learning, suggesting isoform-specific effects on cognitive function^113^. Rab3d KO mice display structural changes in exocrine glands, independent of classical exocytosis pathways^27^.

In *C. elegans*, RAB-3/Rab3 localizes specifically to synaptic vesicles. While RAB-3/Rab3 deficient nematodes are viable, they display subtle behavioral abnormalities, including reduced locomotion, irregular pharyngeal pumping, and atypical defecation cycles^16^. Despite the fact that synaptic vesicle density is significantly diminished, neurotransmitter release is preserved, suggesting a role for RAB-3/Rab3 in vesicle docking or tethering at synaptic release sites^16^. In *D. melanogaster*, Rab3 mutant neuromuscular junctions (NMJs) show an uneven distribution of key presynaptic proteins such as Bruchpilot, calcium channels, and electron-dense T-bars, which are restricted to a subset of active zones^108^. This results in heterogeneity in active zone composition and release properties along axon branches, affecting synaptic function and plasticity^114^.

In age-related neurodegenerative diseases such as Alzheimer’s disease (AD) and Parkinson’s disease (PD), Rab3 expression is diminished^109,115–120^. Based on these converging lines of evidence, it is plausible that Rab3 dysfunction, whether genetic or acquired through disease-related mechanisms, contributes to cognitive and motor impairments. The phenotypes observed in Rab3 mutant models phenocopy key aspects of neurodegenerative disease pathology, supporting Rab3 as a relevant target for understanding disease mechanisms and investigating new pharmacological interventions.

## The role of Rab3 proteins in age-related neurodegenerative pathologies

The Ras superfamily of GTPases, including Rab proteins, has historically been overlooked as a significant factor in the pathogenesis of neurodegenerative diseases^121^. However, growing evidence highlights the potential contributions of various RAB proteins to neurodegeneration, including AD, Parkinson’s disease (PD), and Huntington’s disease (HD). While several reviews have explored the role of Rab proteins in neurodegeneration, the specific involvement of Rab3 proteins remains relatively underexplored^122–125^. Yet, their proper function is essential for maintaining neuronal health, particularly through their established roles in synaptic vesicle trafficking and neurotransmitter release.

Expression levels of Rab3 proteins demonstrate substantial variability across different species, disease models, and experimental methodologies in the context of age-related neurodegenerative pathologies (Table 1). Most studies have reported a downregulation of Rab3 proteins in various neurodegenerative conditions, suggesting that disruptions in Rab3-mediated processes may contribute to disease progression^109,115–120^. Indeed, reduced Rab3 expression is associated with impaired synaptic function, defective neurotransmitter release, and autophagic dysfunction—key pathological features in diseases like AD, PD, and HD. However, a few studies have reported contrasting findings. Specifically, Rab3 proteins were found to be upregulated in the cerebrospinal fluid (CSF) of AD patients^126^ and in the aging brains of Cynomolgus monkeys^127^. In the investigation of CSF biomarkers, in addition to Rab3, the researchers analyzed other Rab proteins, specifically Rab4, Rab5, Rab7, and Rab9^126^. The study revealed that Rab4, Rab5, and Rab9 were undetectable in the CSF when assessed using the Western blot technique^126^. Conversely, Rab3 and Rab7, along with other lysosomal network proteins such as LAMP-1, LAMP-2, and LC3, demonstrated increased concentrations in the CSF of AD patients, indicating their substantial involvement in lysosome-associated processes in the pathology of AD^126^.

**Table 1:** Expression of Rab3 proteins in age-related neurodegenerative diseases

Rab3 isoform	Diseases	Direction	Sample	Test method	References
Rab3a	AD	Down	Human hippocampus and frontal cortex	Western blotting	^120^
Rab3a	AD	Down	Human inferior temporal cortex and inferior parietal cortex	Western Blotting	^119^
Rab3a	AD	Down	Human frontal cortex	Western Blotting	^109^
Rab3a	PDD, DLB, AD	Down	Human prefrontal cortex and inferior parietal lobe neocortex	Western blotting and ELISA	^117^
Rab3a	AD	Down	Human embryonic stem cell-derived Alzheimer’s disease models	Western Blotting	^115^
Rab3a	Diffuse Lewy body disease	Down	Human Entorhinal cortices	Western Blotting	^118^
Rab3	PD	Down	*D. melanogaster* brains	LC-MS/MS	^116^
Rab3	AD	Up	Human Cerebrospinal Fluid	Western Blotting	^126^
Rab3	Aging	Up	Cynomolgus monkey brains	Western Blotting	^127^

These observations suggest a more complex regulatory mechanism, potentially involving feedback loops to compensate for disrupted vesicle trafficking or other cellular stressors. In the aging brains of Cynomolgus monkeys, the upregulation of Rab3 proteins was observed in the context of dynein depletion, which impairs the retrograde transport of vesicles^127^. Interestingly, the elevated Rab3 expression might represent a compensatory response to impaired vesicle trafficking, thereby enhancing synaptic vesicle transport despite defective dynein activity. However, this compensatory mechanism may have unintended consequences. The simultaneous accumulation of lysosomes and LC3-positive autophagic vesicles observed in dynein-depleted cells suggests that increased Rab3 levels might exacerbate traffic congestion, leading to neuritic swelling^127^. These findings highlight a potential link between Rab3 proteins and lysosomal dysfunction, which warrants further investigation.

Rab3 proteins are also implicated in broader cellular processes beyond their canonical role in synaptic vesicle trafficking. Their altered expression levels across neurodegenerative pathologies suggest involvement in mechanisms such as lysosome positioning, autophagy, and organelle interactions—all of which are critical in the context of aging and disease (Table 2). Dysfunction in these pathways could contribute to the accumulation of misfolded proteins, defective organelles, and ultimately, neuronal degeneration. Given the significant alterations in Rab3 protein expression and their impact on neuronal function, understanding the molecular switches regulating Rab3 activity is critical. Their role in maintaining synaptic integrity and their interactions with other organelle systems, such as lysosomes and autophagic pathways, place them at the intersection of key processes implicated in neurodegeneration. Future research should focus on elucidating the precise mechanisms underlying Rab3 protein dysregulation in these diseases, exploring their potential as biomarkers or therapeutic targets.

**Table 2:** Role of Rab3 proteins, Rab3GEF in age-related neurodegenerative diseases

Rab3 isoform	Diseases	Role	Model Used	References
Rab3a	AD	Rab3 is involved in the regulation of trafficking processes and the maintenance of amyloid precursor protein (APP) levels	HEK293 and HeLa cells stably expressing the pathogenic Swedish mutant of APP	^132^
Rab3	AD	Rab3a facilitates the maturation of amyloid precursor protein (APP) transport vesicles, including promoting the recruitment of conventional kinesin.	SH-SY5Y cells and mouse neuroblastoma N2a cells	^131^
Rab3GEF	AD	Rab3-GEF (MADD) functions as a Tau toxicity modulator, and downregulation of Rab3-GEF (MADD) enhances Tau Toxicity.	*D. melanogaster*	^135^
Rab3GEF	AD	DENN/MADD is protective by inhibiting TRADD-induced apoptotic cell death	AD patient hippocampal tissues, APP-overexpressing mice, hippocampal neuron cells	^133^
Rab3GEF	AD	Intact DENN/MADD is required for neuronal survival by protecting against oAβ neurotoxicity	SH-SY5Y cells	^134^
Rab3a	PD	Rab3a confers neuroprotection against α-Synuclein-Induced neuronal Loss.	*C. elegans* and rat primary cells	^149^
Rab3a	PD	The cycle of membrane association and dissociation of α-syn is connected to synaptic activity through the Rab3a recycling mechanism.	PD mouse models	^139^
Rab3a	PD	Rab3a inhibition of Ca^2^-dependent dopamine release from PC12 cells is mediated by its interaction with synaptotagmin I.	PC12 cells	^36^
Rab3b	PD	Rab3B enhances the management and storage capacity of dopamine at presynaptic terminals and provides protection to susceptible dopaminergic neurons.	Rat and DA neuroblastoma cell models	^150^
Rab3	PD	The inactivation of Rab3 facilitates the accumulation of Arl8, potentially implicating a defect in the switching mechanism of retrograde transport at the microtubule plus-end	*D. melanogaster*	^78^
Rab3	PD	Phosphorylation of Rab3a by *LRRK2* interferes with its interaction with the motor adaptor MADD, which may inhibit the assembly of the Rab3a–MADD-KIF1A/1Bβ complex responsible for facilitating anterograde synaptic vesicle precursor (SVP) transport.	Human-induced pluripotent stem cells	^141^
Rab3	HD	The reduction of HTT perturbs the bidirectional axonal transport of vesicles containing Rab3	*D. melanogaster*	^153^
Rab3a	HD	The overexpression of Rab3a mitigates the deficiencies observed in the docking and secretion of BDNF vesicles.	Astrocytes and mouse models	^156^

### Rab3 and AD

Alterations in Rab3a expression have been widely reported in AD, further supporting its involvement in the disease development and progression^117,119,120,128^. Several studies have demonstrated that the levels of Rab3a are diminished in degenerative regions of AD brains, implicating its compromised function in disease. Conversely, elevated Rab3 levels have been reported in the CSF of AD patients^126^. This apparent discrepancy may reflect compensatory mechanisms activated in early stages of the disease, prior to significant synaptic loss. Alternatively, localized decreases in Rab3a levels in specific brain regions may not be fully reflected in CSF measurements.

**Fig. 2:**
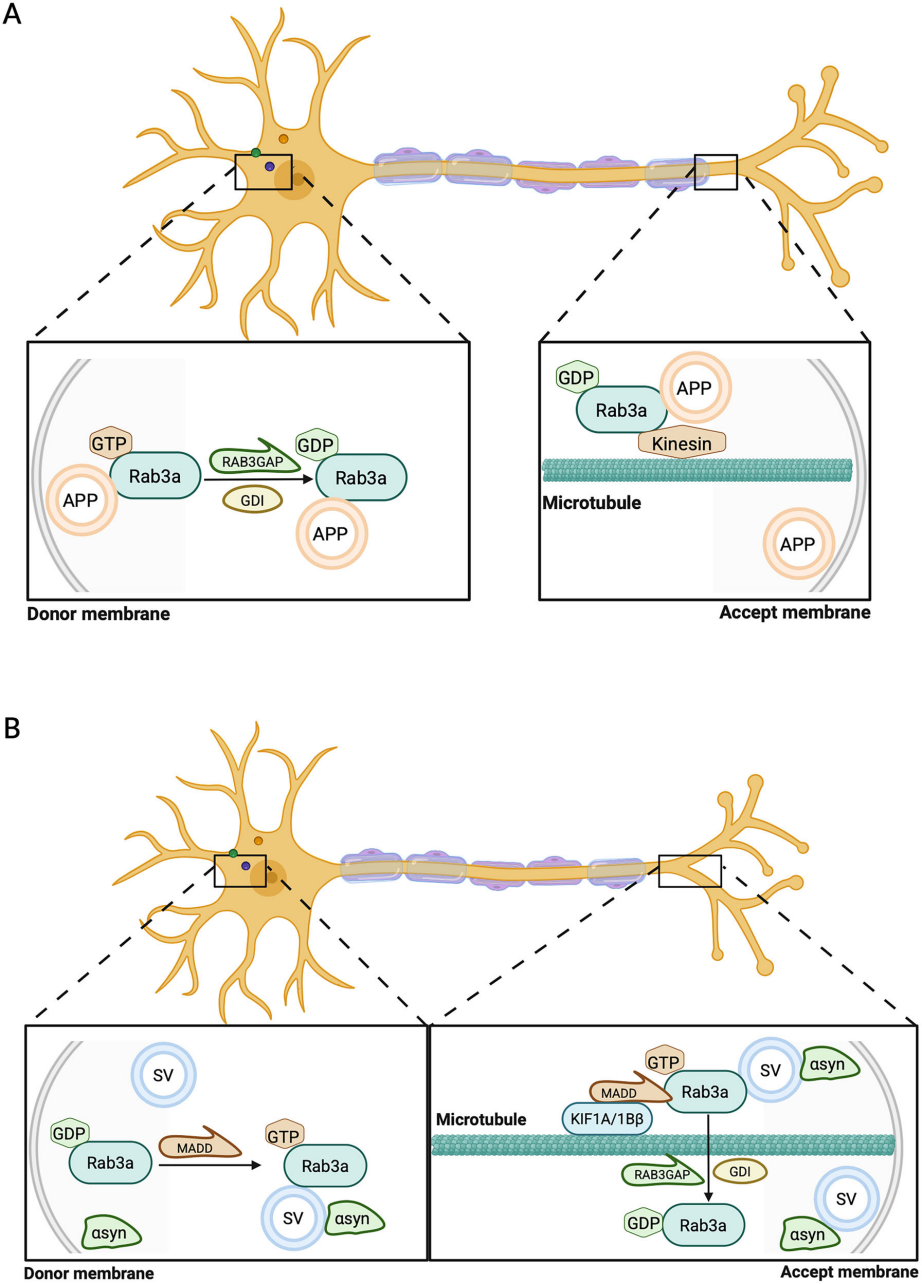
Anterograde transport of APP and αsyn is mediated by Rab3a. **A** In neurons, amyloid precursor protein (APP) is sorted into a fast axonal transport vesicle that includes Rab3a and other presynaptic components. Initially, the GTP-bound form of Rab3a facilitates the packaging and assembly of APP along with a specific set of presynaptic components, such as syntaxin-1, SNAP25, synapsin-1, Munc13-1, and RIM2, within a shared vesicle. Subsequently, the hydrolysis of Rab3a-GTP, catalyzed by dimerized Rab3GAP, induces a conformational transition from Rab3a-GTP to Rab3a-GDP. This transition is accompanied by the recruitment of a specific kinesin-1 isoform variant, kinesin-1C, to the APP transport vesicle. Finally, the assembled vesicle is transported to its target membrane by conventional kinesin along microtubule tracks. **B** The anterograde transport of α-syn is predominantly facilitated by Rab3a in its GTP-bound state rather than GDP-bound state. Upon conversion from the GDP-bound to the GTP-bound form, Rab3a interacts with its effector protein MADD, resulting in the formation of the Rab3a-MADD-KIF1A/1Bβ complex. This complex serves as the driving force for the anterograde transport of synaptic vesicle precursors and α-syn to the presynaptic terminal. The disruption of the interaction between α-syn and Rab3a by GDP dissociation inhibitor (GDI), following the conversion of Rab3a to its GDP-bound state, facilitates the dissociation of α-syn from vesicles. Created in BioRender. Palikaras, K. (2025) https://BioRender.com/t13j594.

Amyloid beta (Aβ) peptides, derived from the proteolytic cleavage of amyloid precursor protein (APP), are the principal components of extracellular plaques characteristic of AD. These peptides exert diverse effects on vesicular transport and neuronal cell function, including disruption of synaptic activity and trafficking pathways^129,130^. APP is transported anterogradely within specialized transport vesicles by conventional kinesin, specifically the kinesin-1C isoform. These vesicles interact with several presynaptic proteins, including Rab3a, which plays a critical role in the assembly and transport of APP to nerve terminals. Rab3a regulates APP transport through its GTPase activity. The conversion of GTP-bound Rab3a to its GDP-bound state facilitates the assembly of kinesin-1C and APP within transport vesicles (Fig. 2A). A functional Rab3a GTPase is essential for the efficient accumulation of APP at nerve terminal ends^131^. Studies have shown that loss of Rab3a GTPase activity or mutations affecting its function can lead to reduced APP transport and accumulation at synapses, impairing normal APP processing^131^. This is particularly relevant because APP can undergo cleavage by α-secretase (ADAM10) within transport vesicles, producing soluble APP (sAPP)^131^. The production of sAPP is thought to mitigate Aβ generation, suggesting that Rab3a’s role in APP transport and processing is crucial for reducing pathogenic Aβ levels. Diminished levels of Rab3a in the brain tissues of AD patients may impair APP transport, leading to its accumulation at synaptic sites. Without cleavage by α-secretase (ADAM10) into sAPP, this buildup of APP can favor the production of pathogenic Αβ peptides. In Rab3a knockout mice, the loss of long-term potentiation (LTP) at hippocampal mossy fiber synapses results in learning and memory deficits^110^, suggesting that Rab3a depletion may similarly exacerbate cognitive impairment in AD.

Beyond Rab3a, other members of the Rab3 family have been identified as crucial regulators in membrane-trafficking events that modulate Aβ production. Notably, silencing Rab3 isoforms, except Rab3c, significantly reduces both Aβ and sAPP levels in non-neuronal cellular models expressing the pathogenic Swedish APP mutation, a mutation linked to familial early-onset AD. These findings suggest that Rab3 proteins modulate APP processing, influencing Aβ generation and plaque formation^132^. Simultaneously, Rab3GEF is crucial for the functionality of Rab3 in Alzheimer’s disease. In Alzheimer’s disease brains, APP-overexpressing mice, and neuronal cells exposed to Aβ, there is a reduction in MADD expression at both the protein and mRNA levels, accompanied by alterations in MADD splicing, which can further promote neuronal cell death^133,134^. Furthermore, studies in *D. melanogaster* suggest that Rab3 proteins may also intersect with Tau pathology^135^. Interestingly, downregulation of Rab3-GEF, the ortholog of MADD and a critical activator of GTP-bound Rab3 proteins, exacerbates Tau toxicity in *D. melanogaster* model^135^. These findings indicate that GTP-bound Rab3 proteins, activated by Rab3-GEF/MADD, play a protective role against Tau-mediated neuronal damage. Such findings point to a dual role of Rab3 proteins in both Aβ and Tau pathologies, highlighting their importance in disease progression.

Similar to how Rab3a deficiency can result in impaired APP transport^131^ and synaptic transmission^32–36^, impaired Rab3 function can also lead to deficiencies in lysosomal transport^73–75^. The potential impact of Rab3a deficiency on autophagy processes, which are crucial for the degradation of excess APP and the maintenance of neuronal health, remains uncertain. Additionally, it is unclear whether the upregulation of Rab3 in cerebrospinal fluid (CSF) serves as a compensatory mechanism or as an indicator of an early stage of disease. Moreover, the mechanisms by which different Rab3 isoforms influence APP processing and Tau toxicity require further investigation. These gaps in our understanding underscore the need to unravel the intricate functions of Rab3 proteins in AD and their potential as therapeutic targets.

### Rab3 and Parkinson’s disease (PD)

Parkinson’s disease (PD) is a progressive neurodegenerative disorder characterized by hallmark motor symptoms, including resting tremor, rigidity, bradykinesia, and postural instability. These symptoms are attributed to the degeneration of dopaminergic neurons in the substantia nigra, resulting in insufficient dopamine levels in the striatum. Pathologically, PD is marked by the accumulation of intracellular protein inclusions known as Lewy bodies, primarily composed of alpha-synuclein (α-syn) and α-syn phosphorylated at serine 129 (pS129), which serve as key biomarkers of the disease^136–138^.

Emerging evidence highlights the intricate relationship between α-syn and the Rab3 protein family, particularly Rab3a, in the context of PD pathogenesis. Rab3a, a small GTPase, is essential for synaptic vesicle trafficking and neurotransmitter release. Studies have demonstrated that α-syn interacts with GTP-bound Rab3a on synaptic vesicles, and this interaction is vital for the membrane association and dissociation cycle of α-syn, a process tightly linked to synaptic activity^139^. Following GTP hydrolysis, the GDP-bound Rab3a dissociates from vesicles, mediated by the coordinated actions of GDP dissociation inhibitor (GDI) and heat shock protein 90 (Hsp90), which also facilitate the release of α-syn from the membrane^139^. Disruptions in this cycle—via inhibition of Rab3a recycling or Hsp90 activity—lead to the pathological accumulation of α-syn on vesicle membranes, contributing to synaptic dysfunction^139^. Conversely, exposure to manganese (Mn) leads to the overexpression of α-syn, which subsequently disrupts the Rab3 cycle^140^. This disruption occurs through the binding of α-syn to GTP-bound Rab3a, inhibiting the interaction between Rab3A-GTP and Rab3-GAP, and consequently obstructing GTP hydrolysis^140^. Notably, reducing α-syn expression can alleviate this disruption, emphasizing the reciprocal relationship between Rab3a and α-syn.

Rab3a also forms a functional complex with its effector protein MADD (MAP kinase-activating death domain protein) and the kinesin motor proteins KIF1A/1Bβ^141^. This Rab3a-MADD-KIF1A/1Bβ complex drives the anterograde transport of synaptic vesicle precursors (SVPs) and α-syn to the presynaptic terminal, ensuring proper synaptic function (Fig. 2B). Mutations in *LRRK2* (leucine-rich repeat kinase 2), particularly the R1441C variant, further implicate Rab3 proteins in PD. Rab3a/b/c/d, as well as other Rab proteins like Rab5a/b/c, Rab8a/b, Rab10, Rab12, Rab29, Rab35 and Rab43, are reported to be specifically phosphorylated by *LRRK2*^80^. This mutation leads to a significant reduction in the expression levels of Rab3a and Rab3-GEF (guanine nucleotide exchange factor) proteins in *D. melanogaster*, disrupting the Rab3 cycle^116^. Phosphorylation of Rab3a by *LRRK2* impairs its interaction with MADD, inhibiting the formation of the Rab3a-MADD-KIF1A/1Bβ complex and consequently disrupting anterograde synaptic vesicle transport^141^. MADD is alternatively referred to as Rab3-GEP (guanine nucleotide exchange protein) because it functions as a guanine nucleotide exchange factor (GEF) for Rab3, facilitating the exchange of GDP for GTP^53^. The phosphorylation of Rab3a hinders MADD’s ability to convert GDP-bound Rab3 into its GTP-bound form, thereby impairing its function in facilitating anterograde synaptic vesicle transport^141^. To demonstrate that impaired Rab3a–MADD interaction inhibits anterograde synaptic vesicle transport, researchers showed that the Q81L mutant Rab3a, which stabilizes Rab3a in a GTP-bound state, counteracted the inhibitory effects of the hyperactive *LRRK2* mutant on SVPs transport^141^. Furthermore, the study revealed that the phosphorylation of Rab3a disrupts its interactions with RAB-GDI1 and Rab3GAP, which function collaboratively to retrieve Rab3 proteins from inappropriate membrane locations^141^. These findings indicate that a hyperactive *LRRK2* mutant would cause dysfunction of Rab3a proteins in facilitating anterograde synaptic vesicle transport^141^. Therefore, hyperactivity of *LRRK2* may reduce the anterograde synaptic vesicle transport of α-syn and other proteins essential for synaptic function. Impairments in the trafficking and targeting mechanisms of α-syn to the appropriate membrane compartment within neurons may affect its tendency to aggregate^142^. α-syn is predominantly localized at the presynaptic terminal, where it engages with the SNARE complex to attenuate synaptic vesicle exocytosis^143,144^. It exhibits a preferential association with synaptic vesicle membranes and lipids in multimeric forms, which serve to inhibit the protein’s transition into β-sheet structures and subsequent inclusion formation^145–148^. Therefore, the dysfunction of Rab3a proteins in cells harboring the *LRRK2* mutation may lead to compromised α-syn transport. When α-syn is not bound to synaptic vesicles and lipids, it may be mislocalized and adopt pathogenic conformations.

Interestingly, Rab3 proteins also exhibit protective effects against α-syn toxicity in various PD models, including *C. elegans*, rat dopamine neurons, and cellular systems^149,150^. Both Rab3a and Rab3b have been shown to mitigate α-syn-induced toxicity, highlighting their potential as therapeutic targets^151^. However, this protective role is isoform-specific and context-dependent. Rab3a negatively modulates dopamine release in PC12 cells, as evidenced by increased dopamine release upon Rab3a knockdown^36^. In contrast, Rab3b enhances dopamine storage capacity and presynaptic regulation in rat dopaminergic neurons and neuroblastoma cell models, while also protecting these neurons from degeneration^150^. Despite these protective roles, an uncontrolled increase in Rab3 protein levels, without the regulatory balance provided by Rab3GAP proteins, could adversely affect synaptic function^36^. Rab3GAP proteins are critical for modulating the Rab3a cycle, ensuring its proper function and preventing disruptions in synaptic vesicle trafficking. Dysregulation of this balance may exacerbate synaptic dysfunction and neuronal damage, complicating the therapeutic application of Rab3 proteins in PD^36,149–151^.

The emerging roles of Rab3 proteins in regulating α-syn dynamics, dopamine storage, and synaptic vesicle transport position them as promising therapeutic targets for PD. However, further research is needed to optimize their application in PD therapy, focusing on maintaining the delicate balance of Rab3 protein activity and their interactions with regulatory partners such as Rab3GAP and MADD. Understanding these complex molecular mechanisms could pave the way for novel interventions to mitigate the progression of PD and preserve neuronal function.

### Rab3 and Huntington disease (HD)

Huntington’s disease (HD) is caused by mutations in the huntingtin (HTT) gene, which results in an expanded CAG trinucleotide repeat encoding a polyglutamine (polyQ) tract within the HTT protein. Individuals with 36 or more CAG repeats are at risk of HD, with longer repeat expansions associated with earlier disease onset. However, the repeat number accounts for only part of the variability in age of onset, with additional environmental, genetic and epigenetic factors influencing disease progression^152^.

A critical pathological hallmark of HD is the loss of functional HTT protein, which disrupts essential cellular processes, including axonal transport. Notably, mutant HTT (mHTT) impairs the bidirectional transport of vesicles containing Rab proteins in *D. melanogaster*, such as Rab3 and Rab19-containing vesicles^153^. Brain-derived neurotrophic factor (BDNF), a neurotrophic factor crucial for neuronal survival and differentiation, is markedly reduced in both HD patients and animal models^154,155^. However, an alternative study reported no significant difference in BDNF levels, both at the protein and transcriptional stages, between astrocytes from the HD mice group and the WT mice group. This finding suggests that mHTT does not influence BDNF production in HD astrocytes^156^. The study further identified that mHTT disrupts the release of brain-derived neurotrophic factor (BDNF) from astrocytes by interacting with GTP-bound Rab3a, consequently inhibiting its interaction with Rab3-GTPase-activating protein (Rab3-GAP). Conversely, the overexpression of Rab3a has been demonstrated to ameliorate the deficits in the docking and secretion of BDNF-containing vesicles in astrocytes from HD mice. This overexpression also results in a reduction of reactive astrocytes in vivo and an improvement in ATP deficiency in vitro^156^. The results suggest that the exchange between GTP and GDP-bound Rab3a is crucial for maintaining stable BDNF secretion in HD astrocytes and ameliorating reactive astrocytes caused by mHTT in HD mice^156^. Therefore, Rab3a may be considered a crucial factor in safeguarding neurons from mHTT and represents a promising therapeutic target for HD. Nevertheless, in contrast to its extensively examined roles in PD and AD, the involvement of Rab3 proteins in HD remains insufficiently explored. Additional research is necessary to elucidate the underlying mechanisms, including the selective binding of mHTT to Rab3a in astrocytes, and to develop effective pharmacological interventions.

## Therapeutic implications and future directions

Many studies about the treatment targeting Rab proteins have been reviewed, providing a comprehensive overview of the current advancements in the pharmacological targeting of Rab proteins^123^. However, studies specifically focusing on therapies targeting Rab3 proteins remain limited. Among pharmacological agents screened for age-related neurodegenerative diseases, only two compounds have been identified as targeting Rab3 proteins: BMS-708163 and Nilotinib. These compounds have been shown to upregulate the expression of Rab3a and SV2B proteins, restoring electrophysiological function in human embryonic stem cell-derived Alzheimer’s disease models^115^. Nilotinib, a leukemia drug, was found to activate the AMPK pathway by inhibiting the phosphatase PP2A, leading to increased AMPK phosphorylation and enhanced autophagy in vivo^157^. Additionally, a recent study on hepatocellular carcinoma demonstrates that AMPK pathway activation stabilizes Rab3d mRNA, resulting in increased Rab3d expression^158^. These findings indicate that Rab3 protein homeostasis may be regulated via the AMPK pathway, linking Rab3 to autophagy and lysosomal function. Although these studies involve different disease contexts and tissue types, they suggest that Nitotinib’s influence on Rab3 expression may be isoform- and tissue-specific. The apparent absence of Rab3d upregulation in neuronal models, combined with its known enrichment in non-neuronal tissues, raises the possibility that nilotinib’s effects on Rab3d may be more relevant outside the nervous system. Further studies are needed to clarify the tissue-specific regulation of Rab3 isoforms in response to nilotinib and the potential role of the AMPK-autophagy axis in this process.

Future studies should focus on the investigation of Rab3 proteins in the context of age-related neurodegenerative diseases, particularly by leveraging drugs that activate the AMPK pathway. Autophagy, a lysosome-mediated catabolic process responsible for recycling cellular components and removing damaged organelles, is critical for neuronal health. Neurons are particularly vulnerable to disruptions in the autophagy-lysosomal system, especially with aging, and mutations in genes regulating these processes can contribute to neurodegenerative diseases^159^. Given the importance of Rab3 proteins in exocytosis and synaptic functions, it is crucial to investigate their potential roles in lysosomal activity and autophagy. A better understanding of Rab3 proteins in these pathways could pave the way for novel therapeutic interventions targeting age-related neurodegenerative pathologies.

## Conclusion

The Rab3 family proteins play crucial roles in maintaining synaptic integrity and function, with their dysregulation evidenced in several age-related neurodegenerative diseases. Understanding the molecular mechanisms by which Rab3 proteins regulate neuronal homeostasis and influence cell survival is essential to developing Rab3-related innovative therapeutic strategies. As the prevalence of neurodegenerative disorders continues to rise in aging populations, it is urgent to develop effective therapeutic strategies, such as via modulating protein activities and the use of artificial intelligence (AI) in fast screening drug candidates^160–162^. Among the protein targets, it seems that Rab3 family proteins are potential candidates as their activities can be modulated pharmaceutically. Notably, Nilotinib can upregulate Rab3a expression and activate the AMPK pathway, which may further regulate Rab3 function^115,157,158^. These findings suggest that other pharmacological agents capable of activating AMPK may also influence the activities of Rab3 proteins. Further biochemical, molecular, and cellular studies focusing on the functions of Rab3 family proteins, as well as the development of Rab3 activators, are needed.

## Data Availability

No datasets were generated or analysed during the current study.
